# Personal

**Published:** 1892-05

**Authors:** 


### Personal.

Dr. E. E. Montgomery, Professor of Gynecology in the Medico-Chirurgical College of Philadelphia, has been elected Professor of Clinical Gynecology in Jefferson Medical College. This is another evidence that Jefferson is making a fight for the supremacy, and she appears destined to resume her old place as a strong and well-equipped medical school. Dr. Montgomery has demonstrated the fact that he is one of the best teachers of clinical gynecology in the country, and Jefferson is to be congratulated in securing him for that chair.

Dr. J. L. Eddy, of Olean, one of the most prominent physicians of the southern tier, has been appointed aide-de-camp on the staff of the State Commander of the Grand Army of the Republic. This is a fitting recognition of Dr. Eddy's military service, and of his devotion to the interests of soldiers.

Dr. A. A. Hubbell, Professor of Ophthalmology in Niagara University, sailed for Europe in the early days of April. He will enjoy a two months' vacation, visiting the important clinics of Europe, and return about June 1st, in season to participate in the Summer society meetings.

Wm. C. Krauss, M. D., of Buffalo, has been appointed associate editor on the staff of *Neurologisches Centralblatt*, published by Prof. E. Mendel, of Berlin, Germany.

Dr. F. J. Thornbury, formerly resident physician at the Cincinnati Hospital, has permanently located in Buffalo, and established his office at 610 Main street. Dr. Thornbury has lately spent some months in Europe engaged in bacteriological studies, and is well equipped for the practice of his profession.

Errata.—Page 586 : *has* instead of had, tenth line from the bottom. Page 587 : *achromatopsia* instead of actromatopsia, tenth line from the bottom. Page 588 : *regurgitant* instead of engurgitant, eighteenth line from the bottom.

The selection on page 619, Medico-Legal and Medico-Ethical, Denholm vs. Tait, is from the *British Medical Journal*, April 2, 1892.

